# Supplementary material for: A Zn(II)–Metal–Organic Framework Based on 4-(4-Carboxy phenoxy) Phthalate Acid as Luminescent Sensor for Detection of Acetone and Tetracycline
Source: Molecules. 2023 Jan 19;28(3):999. doi: 10.3390/molecules28030999 (PMC9921817; doi:10.3390/molecules28030999)
Supplement: Supplementary file 1 [file molecules-28-00999-s001.zip › molecules-2162571-supplementary .pdf]

molecules

Article

A Zn(II)–Metal–Organic Framework Based on 4-(4-Carboxy phenoxy) Phthalate Acid as Luminescent Sensor for Detection of Acetone and Tetracycline

Nairong Wang , Shanshan Li , Zhenhua Li , Yuanyuan Gong , Xia Li*

Department of Chemistry, Capital Normal University, Beijing 100048, China; wnr1995@163.com (N.W.); 2210702011@cnu.edu.cn (S.L.); 2190702005@cnu.edu.cn (Z.L.); 2210702007@cnu.edu.cn (Y.G.)
* Correspondence: xiali@cnu.edu.cn

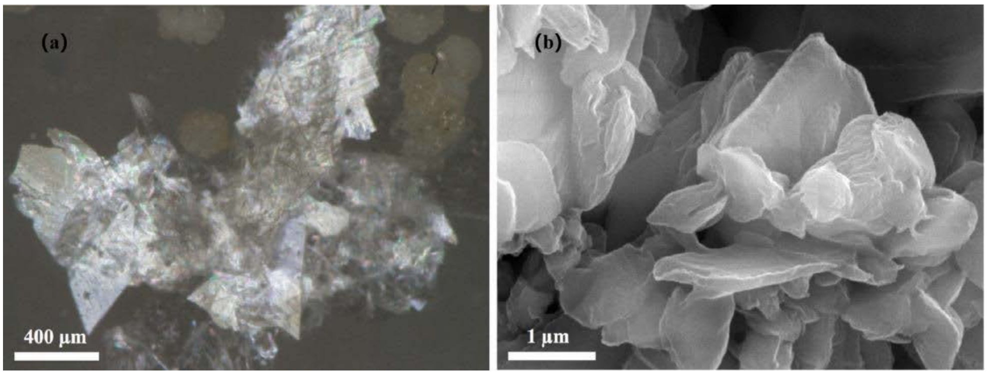

Figure S1. (a) Optical microscopy image and (b) SEM image of the Zn-MOF.

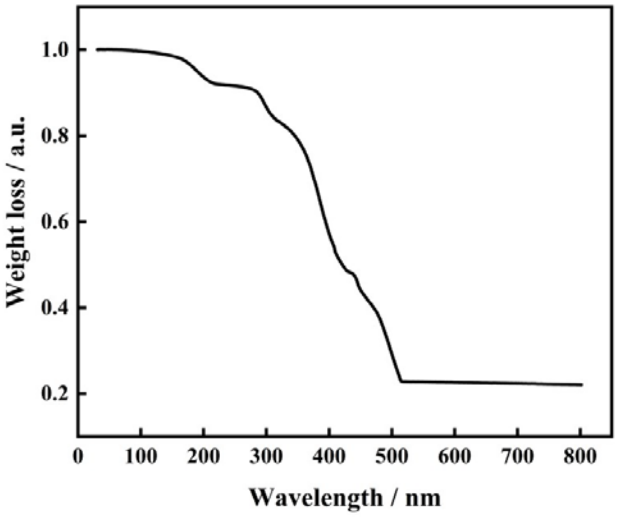

Figure S2. TGA curve of Zn-MOF.

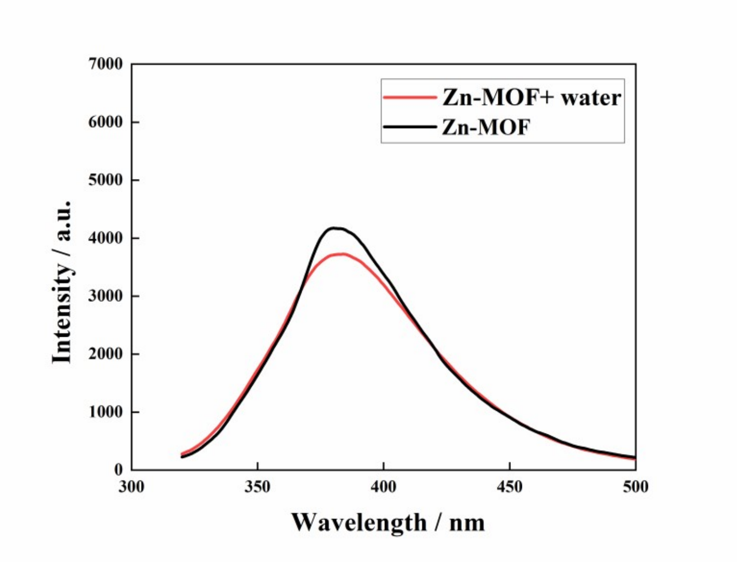

Figure S3. The emission spectra of Zn-MOF in solids and water.

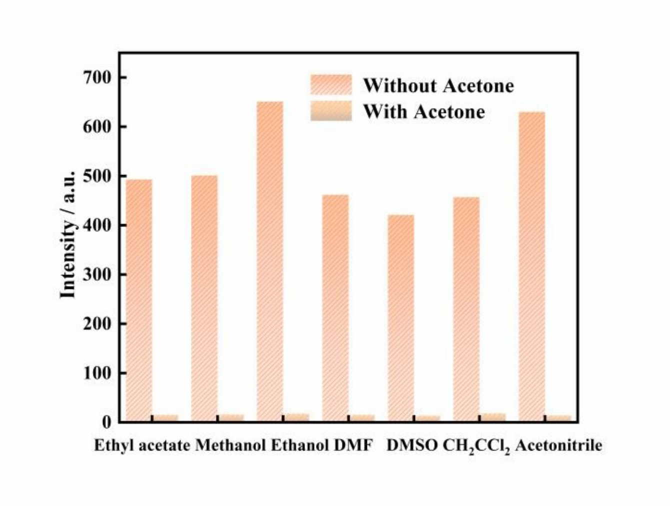

Figure S4. The luminescence intensity of Zn-MOF in different interfering solvents with and without acetone.

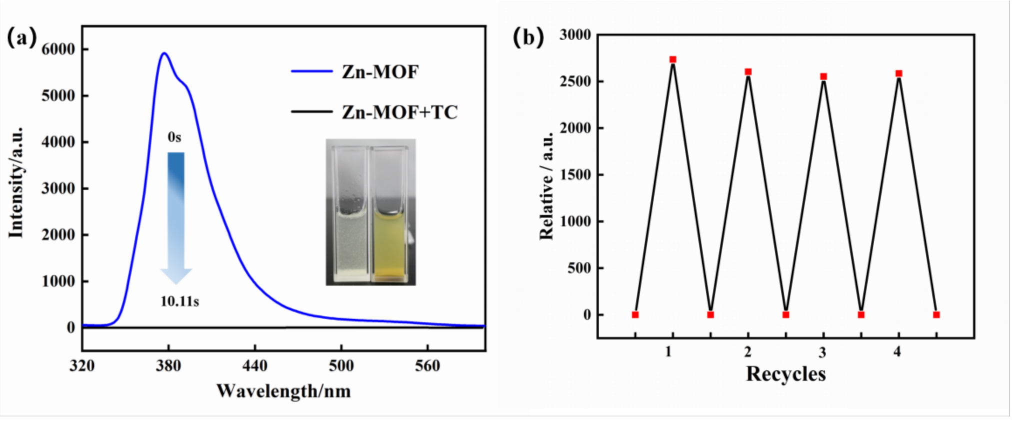

Figure S5. (a) Time response of Zn-MOF to TC and (b) recycling experiment.

Table S1 The crystal data of Zn-MOF.

Complex	**1**
Empirical formula	$C_{52}H_{44}Zn_3N_{10}O_{20}$
Formula weight	1325.08
Crystal system	Monoclinic
Space group	$P2_1/n$
a/Å	7.8505(4)
b/Å	15.8782(6)
c/Å	21.6811(8)
α/°	90
β/°	90.08
γ/°	90
Volume (Å^3)	2702.6(2)
Z	2
Calculated density g/cm^3	1.628
Absorption coefficient /mm^{-1}	1.410
F (000)	1352
Crystal size/mm^3	0.461 x 0.113 x 0.107
Theta range for data collection/(°)	2.895 to 27.555
Limiting indices	$-9 \le h \le 10$,
Reflections collected/ unique	26989/ 4851
Data/restraints/parameters	4851 / 0 / 394
Goodness-of-fit on F^2	1.058
Final R indices [$I > 2\sigma(I)$]	R1=0.0431,
R Indices (all data)	R1= 0.0815,
Largest diff. peak and hole/e Å^{-3}	0.432 and -0.328
CCDC No.	2167668

Table S2 Selected bond lengths [Å] and angles [°] for Zn-MOF.

Zn-MOF			
Zn(1)-O(4)A	1.946(2)	Zn(2)-O(8)	2.118(2)
Zn(1)-O(2)	1.933(2)	Zn(2)-O(8)A	2.118(2)
Zn(1)-N(1)	2.024(2)	Zn(2)-O(9)	2.079(2)
Zn(1)-N(5)	2.010(3)	Zn(2)-O(9)A	2.079(2)
Zn(2)-N(3)A	2.140(2)	Zn(2)-N(3)	2.140(2)
O(2)-Zn(1)-O(4)A	125.85(11)	O(9)B-Zn(2)-O(8)B	94.96(8)
O(2)-Zn(1)-N(1)	100.22(11)	O(9)-Zn(2)-O(8)	94.96(8)
O(2)-Zn(1)-N(5)	95.94(11)	O(9)-Zn(2)-O(8)B	85.04(8)
O(4)A-Zn(1)-N(1)	110.10(9)	O(9)B-Zn(2)-O(8)	85.04(8)
O(4)A-Zn(1)-N(5)	112.76(9)	O(9)B-Zn(2)-O(9)	180
N(5)-Zn(1)-N(1)	110.53(10)	O(9)-Zn(2)-N(3)	88.23(9)
O(8)-Zn(2)-O(8)B	180	O(9)B-Zn(2)-N(3)	91.76(9)
O(8)B-Zn(2)-N(3)	90.97(10)	O(9)-Zn(2)-N(3)B	91.77(9)
O(8)B-Zn(2)-N(3)B	89.03(9)	O(9)B-Zn(2)-N(3)B	88.24(9)
O(8)-Zn(2)-N(3)B	90.97(10)	N(3)-Zn(2)-N(3)B	180
O(8)-Zn(2)-N(3)	89.03(9)		

Symmetry transformations used to generate equivalent atoms:

A: -x-3/2,y+1/2,-z+1/2 B: -x-3,-y,-z+1 C: -x-7/2,y+1/2,-z+1/2 D: -x-3/2,y-1/2,-z+1/2 E: -x-7/2,y-1/2,-z+1/2
